# Factors influencing the health-seeking behavior of Vietnamese migrants in Japan: a cross-sectional study on knowledge, attitudes, and practices towards tuberculosis

**DOI:** 10.1186/s41182-025-00766-w

**Published:** 2025-06-18

**Authors:** Sangnim Lee, Nhan Nguyen Huu Thanh, Yusuke Akutsu, Yoshihisa Shirayama, Pham Nguyen Quy, Jin Takasaki, Akihiro Ohkado

**Affiliations:** 1 Department of Epidemiology and Clinical Research, the Research Institute of Tuberculosis, Japan Anti-Tuberculosis Association, 3-1-24 Matsuyama, Kiyose City, Tokyo, 204-8533 Japan; 2 Department of Respiratory Medicine, National Center for Global Health and Medicine, Tokyo, Japan; 3 Department of Global Health Research, Graduate School of Medicine, Juntendo University, Tokyo, Japan; 4 Migrant Health Action Network (MiHAN), Tokyo, Japan; 5 Department of Medical Oncology, Kyoto Miniren Central Hospital, Kyoto, Japan

**Keywords:** Tuberculosis, Foreign-born, Migrant, Knowledge, Attitudes, Practices, Risk communication, Behavior

## Abstract

**Background:**

Addressing tuberculosis (TB) among migrants from high-burden countries is important for the health of migrants and for public health in low-TB-burden countries. Therefore, approaches that enable migrants to access TB diagnostic services and care early are required. To develop TB risk communication for migrants from high-TB-burden countries, this study aimed to assess Vietnamese migrants’ knowledge, attitudes, and practices (KAP) towards TB and its association with health-seeking behaviors.

**Methods:**

A cross-sectional study was conducted among Vietnam-born migrants aged 18 years and older in two cities in Japan. A self-administered online survey consisted of questions on demographics, health-related issues and behaviors, and the KAP towards TB. Participants who would not seek healthcare even if they had TB symptoms were categorized as having "non-health-seeking behavior", and related factors were examined using multiple logistic regression analysis.

**Results:**

A total of 230 Vietnamese migrants participated in this study. Technical intern trainees (46.1%) and workers (28.7%) comprised the majority of the participants. Overall, 73.9% believed that persons infected with TB were infectious, and 46.1% reported concerns about being diagnosed with TB. Their concerns included maintaining employment and continuing schooling during treatment. Ten percent of the participants stated that they would not consult a doctor even if they developed TB symptoms. Multiple logistic regression analysis revealed that participants who believed that TB could not be cured were significantly more likely to exhibit non-health-seeking behavior (adjusted odds ratio: 3.12, 95% confidence interval 1.14–8.52) compared to those who believed TB could be cured.

**Conclusions:**

Tailored TB risk communication should address migrants’ misconceptions and concerns regarding TB in the host countries. Further efforts are needed to improve TB knowledge through TB education and to disseminate information before and after migration. Creating a supportive environment, such as language assistance and work- and school-related social support, is also needed to facilitate the early detection of TB and healthcare access among migrants.

## Background

Tuberculosis (TB) is preventable and curable; however, it remains one of the world’s top infectious killers and a disease that often affects vulnerable people. The implementation of the World Health Organization’s END TB Strategy requires efforts from all countries [1]. Managing TB among migrants from high-burden countries is important for the health of migrants and for public health, and various initiatives are being implemented in low TB-burden countries such as pre- and post-migration TB screening [2, 3], and cross-border collaboration for patients’ continued TB care [4, 5]. Japan became a low-TB-burden country in 2021; however, the proportion of foreign-born cases among the newly notified TB patients has continued to rise, reaching 16.0% in 2023 [6]. Among newly notified TB cases in their 20 s, foreign-born cases accounted for 84.8%. According to a TB statistics report in Japan, “patient delay” is defined as the time between the onset of symptoms and the initial doctor visit. In 2023, the proportion of foreign-born pulmonary TB patients with patient delays longer than one month (37.6%) was higher than that of Japan-born patients (30.9%) [6]. Due to the increasing trend of foreign-born newly notified TB patients, Japan Pre-entry TB Screening (JPETS) has commenced gradually, starting with some target countries since 2025, and Vietnam is included among these target countries [7, 8].

Vietnam-born individuals were the largest group of newly notified overseas-born TB cases in 2019 and the second largest since 2018. Strengthening TB responses among Vietnamese migrants in Japan is important because they have also represented the second-largest group of foreign residents in recent years. More than half of the Vietnamese residents are on work-related permits, which generally allow them to stay in Japan for less than five years [9], making them highly mobile. Additionally, social vulnerabilities have recently been observed among young adult Vietnamese migrants with TB, including language barriers, limited access to social support for TB-related issues, and difficulties in accessing TB care during the COVID-19 pandemic [10–13]. Efforts are needed to ensure that migrants have early access to TB diagnostic services and care to help mitigate the challenges surrounding TB in the host country.

When addressing the needs of these key populations in infectious disease control, it is often difficult for health authorities to adequately reach migrants because of language and cultural differences and a limited understanding of communication channels. Therefore, it is crucial to strengthen risk communication on infectious diseases for migrants by addressing the needs and social barriers of the target group [14, 15]. Our preliminary study conducted to develop TB risk communication reported that recent migrants from Vietnam often demonstrated health-seeking behaviors, such as relying primarily on social networking services to seek health information and advice in Japan [11]. However, limited information is available regarding the KAP-related factors influencing healthcare access for TB symptoms among migrants in Japan. To develop risk communication for Vietnamese migrants, this study aimed to describe the knowledge, attitudes, and practices (KAP) towards TB among Vietnamese migrants in Japan and to assess their association with health-seeking behaviors.

## Methods

### Study design and setting

We conducted a cross-sectional survey on TB-related KAP and health-seeking behaviors among Vietnamese migrants in Tokyo and Osaka, where there is a large population of Vietnamese residents, as part of a risk communication development research program from April 2022 to March 2026. This program aimed to develop evidence-based risk communication strategies for Vietnamese migrants in Japan and employed participatory action research (PAR). The program was conducted in collaboration with the Vietnamese migrant community throughout the research process. Human resources were mobilized from the Vietnamese migrant community, who volunteered as members of a nonprofit network called the Migrant Health Action Network (MiHAN). Following the survey, the study participants were offered free migrant-friendly health consultations provided by MiHAN medical volunteers at the survey venues according to their needs.

### Study population and data collection procedures

Vietnamese migrants aged 18 years and older who attended large-scale cultural festivals in June 2023 were invited to participate in a questionnaire survey. Participants were informed of the study purpose, their confidentiality, and their right to withdraw at any time before completing the survey. Only those, who provided written informed consent via an online survey form, participated in the self-administered online survey using internet devices. Record of informed consent was stored in an online database.

### Measures

A TB-related KAP and health survey was developed based on a literature review [16]. TB knowledge questions were carefully selected by the research team to suit the general public. The “practice” component included questions to assess health-seeking behaviors among participants. In this study, we defined “health-seeking behavior” as any action taken by individuals to seek healthcare services when experiencing health problems [17].

The English version of the questionnaire was then translated into Vietnamese. The translated version was reviewed through back-translation to ensure accuracy. Pilot testing was conducted with five Vietnamese volunteers, and the questionnaires were revised based on their feedback to ensure clarity and relevance to Vietnamese understanding. The self-administered online questionnaire consisted of the following components: (1) demographics, (2) health-related issues and behaviors, and (3) KAP towards TB. To broadly understand the participants’ health conditions and behaviors, the questionnaire included questions related to common TB symptoms such as cough, phlegm, fever, difficulties with breathing, fatigue, weight loss, and bloody phlegm. Additionally, questions regarding preferred sources of information were included to explore the development of risk communication programs.

### Data analysis

The main variables are presented in frequency tables using descriptive statistics. In this study, responses to the practice-related question, “If you had the following symptoms of TB (e.g., you had a cough for three weeks or longer or if you were coughing up blood in your sputum), at what point would you go to a medical facility?” was used as the key indicator of “health-seeking behavior”. The response “I would not go to see a doctor” was defined as “non-health-seeking behavior”. All other responses, such as “when treatment on my own does not work”, “when symptoms that look like TB signs last for 3–4 weeks”, and “as soon as I realize that my symptoms might be related to TB” were combined and defined as “health-seeking behavior”. In this study, the following occupation or social position of participants were recategorized; worker includes “office worker”, “health personnel or long-term care worker”, and “teacher or researcher”; student includes “Japanese language students” and “university students”; other includes self-employed or freelancer, household worker, and other.

Variables related to knowledge and attitudes towards TB that may lead to the indicator were selected, and a univariate logistic regression analysis was used to estimate the association between these variables and non-health-seeking behaviors. Multiple logistic regression analysis was performed to estimate the odds ratios (OR) of non-health-seeking behaviors. Adjusted OR (aOR) was calculated with a 95 percent confidence interval (95% CI), and a *p-*value of less than 0.05 was considered statistically significant. All the analyses were performed using Stata version 16.1 (StataCorp, College Station, TX, USA).

## Results

A total of 230 Vietnamese migrants participated in this study. Of these, 66.9% were in their twenties. Technical intern trainees (46.1%) and workers (28.7%) comprised the majority of participants. Participants lived in 19 prefectures across Japan (Table 1). Table 2 describes the health-related issues and behaviors of the respondents. Of these participants, 23.0% reported TB-like symptoms and 10.9% reported respiratory symptoms.

**Table 1:** Demographics of the Vietnamese participants

Category	Subcategory	*n*	%
Sex	Female	131	57.0
	Male	98	42.6
	Others	1	0.4
Age group (years)	18–19	3	1.3
	20–29	154	66.9
	30–39	67	29.1
	40–49	6	2.6
Occupation or social position	Worker*	66	28.7
	Technical intern trainee	106	46.1
	Student**	39	16.9
	Unemployed	6	2.6
	Others***	13	5.7
Resident area****	East Japan	129	56.0
	West Japan	101	44.0
Do you have health insurance in Japan?	Yes	217	94.4
	No	12	5.2
	Other	1	0.4
Educational background	Junior high school or lower*****	7	3.0
	High school	74	32.2
	College and professional school	59	25.7
	Higher education (undergraduate, postgraduate)	88	38.3
	Other	2	0.9
What is your Japanese language ability level? (Level by the Japanese-Language Proficiency Test)	Native level or very well (Equivalent to N1)	25	10.8
	Well (Equivalent to N2)	41	17.8
	Intermediate (Equivalent to N3)	66	28.7
	Basic (Equivalent to N4)	79	34.4
	Nonfluency in Japanese	19	8.3
Length of stay in Japan******	Less than 1 year	34	14.8
	1–4 years	123	53.5
	5 years and over	73	31.7

The number of participants was 230. Data were collected at Tokyo (*n* = 127) and Osaka (*n* = 103). *Worker includes office worker (54), health personnel or long-term care worker (11), and teacher or researcher (1). **Student includes Japanese language students (32) and university students (undergraduate or postgraduate) (7). ***Other includes self-employed or freelancer (2), household worker (2), and other (9). ****The residents were from 19 prefectures (East Japan, 56%; West Japan, 44%). *****Junior high school or lower includes junior high school (1), elementary school (4), and no school (2). ******Answers to "4–5 years" were not collected, owing to the statement being incorrectly presented

**Table 2:** Health-related issues and behaviors of the Vietnamese participants

Category	Subcategory	*n*	%
Do you have any of the following symptoms? (1) Cough lasting more than 2 weeks, (2) Phlegm lasting more than 2 weeks, (3) Fever (including slight fever) lasting more than 2 weeks, 4) Difficulties with breathing lasting more than 2 weeks, (5) Fatigue lasting more than 2 weeks, (6) Weight loss of more than 3 kg within 1 year, (7) Bloody phlegm	Yes	53	23.0
	No	177	77.0
If you answer "yes" in question above, please select symptoms that you have (Check all that apply) (*n* = 53)*	Cough lasting more than 2 weeks	15	6.5
	Phlegm lasting more than 2 weeks	8	3.5
	Fever (including slight fever) lasting more than 2 weeks	5	2.2
	Difficulties with breathing lasting more than 2 weeks	4	1.7
	Fatigue lasting more than 2 weeks	9	3.9
	Weight loss of more than 3 kg within 1 year	18	7.8
	I have ever had bloody phlegm	6	2.6
This question is for someone with any of the symptoms listed in question above. When you had these symptoms, did you go to a medical facility in Japan? (Check only one that applies) (*n* = 53)	Yes, I went to a medical facility	22	41.5
	No, I did not go to a medical facility	31	58.5
If you answered “no” in the question above, please indicate why you did not go to a medical facility in Japan. (Check only one that applies) (*n* = 31)	Did not think it was serious	6	19.4
	Worried about the cost	0	0.0
	Language barrier (Japanese)	5	16.1
	Unable to apply for leave from work/school**	0	0.0
	Did not want to find out that I have a disease	0	0.0
	Did not know which medical facility to go to	1	3.2
	Other reasons ***	2	6.5
	No answer	17	54.8
Do you have any health problems?	Yes	39	17.0
	No	191	83.0
When last did you have a health check-up including a chest X-ray in Japan?	This year (2023)	94	40.9
	Last year (2022)	85	37.0
	2021 or before	12	5.2
	Never had a health check-up in Japan	39	17.0
How often do you generally seek health care at a clinic or hospital in Japan? (Check only one that applies)	Regularly (e.g., every month)	13	5.7
	2 times per year or more	49	21.3
	Once a year	132	57.4
	Once in the past 5 years	6	2.6
	Never in the past 5 years	30	13.0
How often do you smoke?	Smoke everyday	19	8.3
	Smoke, but not everyday	9	3.9
	Former smoker, but have quit smoking for more than a month	11	4.8
	Never smoked	191	83.0
How often do you drink per day?	Drink more than 60 g of alcohol (e.g., 3 cans of beer) per day everyday	12	5.2
	Drink more than 60 g of alcohol (e.g., 3 cans of beer) per day but not everyday	14	6.1
	Occasionally drink less than 60 g of alcohol (e.g., 3 cans of beer) per day	90	39.1
	I don’t drink alcohol	114	49.6

The number of participants was 230. *10.9% (25/230) of the participants had respiratory symptoms (having either "cough lasting more than 2 weeks" or "phlegm lasting more than 2 weeks" or "difficulties with breathing lasting more than 2 weeks" or "I have ever had bloody phlegm"). **Although this question required the selection of only one category, one person selected this category along with another category (language barrier), so we used the first category selected by this person as the formal answer. ***Reasons given included the sore throat was due to tonsillitis (1) and an unclear answer (1)

### TB-related knowledge and attitudes

Overall, 33.0% of the participants reported that they had previously learned about TB, and 73.9% believed that people infected with TB could transmit TB bacilli to others. Seventy percent identified “through the air” as the mode of transmission. Eighteen percent believed that TB was incurable, while 56.5% expressed a fear of getting sick because of TB. Seventy percent reported that they did not have anyone to consult if they were diagnosed with TB in Japan. The majority of the participants indicated that they would not talk to anyone other than their family members about the illness if they were sick with TB. Fifty-seven percent responded “I feel compassion and desire to help.” regarding individuals with TB, indicating supportive attitudes towards people with TB. Furthermore, 46.1% indicated that they would worry if they were diagnosed with TB in Japan. The main reason for this was to maintain a job or school while undergoing treatment (Table 3).

**Table 3:** Knowledge and attitudes towards TB among the Vietnamese participants

Category	Subcategory	*n*	%
*Knowledge*			
Have you ever learned about tuberculosis?	Yes	76	33.0
	No	154	67.0
If your answer "yes" in the previous question, where did you first learn about tuberculosis? (Check only one that applies) (*n* = 76)	In Vietnam	62	81.6
	In Japan	12	15.8
	Other countries	0	0.0
	No answer	2	2.6
What are the signs and symptoms of pulmonary tuberculosis? (Check all that apply)	Cough	155	67.4
	Cough that lasts longer than 3 weeks	136	59.1
	Coughing up blood	124	53.9
	Weight loss	105	45.7
	Fever	71	30.9
	Fever without a clear cause that lasts more than 7 days	80	34.8
	Shortness of breath	112	48.7
	Ongoing fatigue	99	43.0
Do you think you can transmit tuberculosis bacilli to others just by being infected?	Yes	170	73.9
	No	60	26.1
How can a person get infected with tuberculosis bacilli? (Check all that apply)	Through handshakes	30	13.0
	Through the air when an infected person coughs or sneezes	161	70.0
	Through sharing dishes	84	36.5
	Through touching items in public places (doorknobs, handles in transportation, etc.)	53	23.0
	Do not know	42	18.3
How can a person avoid getting infected with tuberculosis bacilli? (Check all that apply)	Covering mouth and nose when coughing or sneezing	178	77.4
	Avoid sharing dishes	108	47.0
	Closing windows at home	25	10.9
	Through good nutrition	99	43.0
	Do not know	30	13.0
Can tuberculosis disease be cured?	Yes	189	82.2
	No	41	17.8
How can someone with tuberculosis disease be cured? (Check only one that applies)	Specific drugs given by medical facilities	182	79.1
	Over-the-counter drugs	4	1.7
	Home rest without medicine	2	0.9
	Herbal remedies	7	3.0
	Do not know	35	15.2
	Others	0	0.0
*Attitudes*			
What would your reaction be if you found out that you got sick because of tuberculosis? (Check all that apply)	Fear	130	56.5
	Surprise	42	18.3
	Embarrassment	9	3.9
	Sadness or hopelessness	30	13.0
	Nothing in particular	57	24.8
Do you have anyone you can consult with if you were diagnosed with tuberculosis in Japan?	Yes	69	30.0
	No	161	70.0
Who would you talk to about your illness if you fall ill with tuberculosis? (Check all that apply)	Close friend	40	17.4
	Family members and relatives	104	45.2
	Workplace staff	28	12.2
	Supervising organizations or training staff under the technical intern training program	33	14.4
	School teacher or staff	18	7.8
	Others	1	0.4
	No answer	70	30.4
Which statement best describes your feelings about people with tuberculosis disease? (Check only one that apply)	“I feel compassion and desire to help.”	132	57.4
	“I feel compassion but tend to stay away from these people.”	14	6.1
	“I fear them because they may infect me.”	34	14.8
	“I have no particular feeling towards them.”	50	21.7
Is there anyone you know who has/had tuberculosis? (Check all that apply)	Yes, in Vietnam	57	24.8
	Yes, in Japan	15	6.5
	Yes, in other countries	6	2.6
	No	154	67.0
In Vietnam, how is a person with tuberculosis usually regarded/treated? (Check only one that apply)	The community mostly supports and helps him or her	99	43.0
	Most people are friendly, but they generally try to avoid him or her	43	18.7
	Most people reject him or her	14	6.1
	I do not know	69	30.0
	Others	5	2.2
Is there anything to worry about if you are diagnosed with tuberculosis in Japan?	Yes	106	46.1
	No	124	53.9
If you answered "yes" in the previous question, what would worry you the most if you are diagnosed with tuberculosis in Japan? (Check only one that applies) (*n* = 106)	To maintain work or school during treatment	49	46.2
	TB infection may make it difficult for me to stay in Japan (e.g., visa application)	17	16.0
	Medical expenses	15	14.2
	My Japanese ability when communicating with medical staff during diagnosis and treatment	8	7.6
	Being hospitalized for treatment if infected	11	10.4
	Being discriminated against	3	2.8
	Others	2	1.9
	Missing	1	0.9

The number of participants was 230

### Practices: health-seeking behaviors and sources of information

Most participants reported willingness to access healthcare services if they experienced TB symptoms. However, 24 participants (10.4%) indicated that they would not seek healthcare services. The major reasons cited were concerns about medical costs (33.3%) and language barriers when communicating with medical personnel (29.2%). The percentage of participants who trusted health information from the government (48.7%) and medical professionals (28.7%) was higher than that from any other media, while news on Vietnamese social media, including Facebook (66.1%), was identified as the most effective source of information on TB for reaching Vietnamese people (Table 4).

**Table 4:** Practices, health-seeking behaviors, and sources of information among the Vietnamese participants

Category	Subcategory	*n*	%
*Practices: Health-seeking behaviors*			
If you had the following symptoms of tuberculosis (e.g., You had a cough for more than 3 weeks or if you were coughing up blood in your sputum), at what point would you go to a medical facility? (Check only one that applies)	When treatment on my own does not work	53	23.0
	When symptoms that look like tuberculosis signs last for 3–4 weeks	53	23.0
	As soon as I realize that my symptoms might be related to tuberculosis	100	43.5
	I would not go to a medical facility	24	10.4
If you selected “I would not go to the medical facility” in the previous question, what is the reason? (Check only one that applies) (*n* = 24)	Worry about medical costs	8	33.3
	Language barriers in communicating with Japanese medical personnel in Japanese	7	29.2
	Cannot leave work or school (e.g., work hours overlap with medical facility working hours)	1	4.2
	Do not want to find out that something is really wrong	2	8.3
	Others	3	12.5
	No answer	3	12.5
What help would you seek, if you thought you had symptoms of tuberculosis in Japan? (Check all that apply)	Information provision about the medical facilities where I should visit for appropriate medical services	155	67.4
	Language assistance of medical interpreters in communicating with medical staff	70	30.4
	Consultation about medical costs	58	25.2
	Someone who can accompany me to visit appropriate medical services	31	13.5
	I would not seek help	5	2.2
	Others	11	4.8
If a chest X-ray examination is provided free of charge this year, are you willing to receive it? (Check only one that applies)	Yes, I would to take it	117	50.9
	Yes, I would take it especially if I can receive health consultations free of charge	18	7.8
	No, because I receive annual health check-ups	46	20.0
	No, because I do not have any symptoms now	11	4.8
	No, because I am reluctant to take it	11	4.8
	No, because I am afraid a health problem may be found	27	11.7
*Sources of information*			
Which source of health information do you trust the most? (Check only one that applies)	Published by the government	112	48.7
	Published by a medical professional	66	28.7
	TV	13	5.7
	Social media	19	8.3
	Newspapers and magazines	11	4.8
	Others	9	3.9
Please indicate what information you would like about tuberculosis (Free descriptions)	*Please see the footnote for answers		
Which sources do you think can most effectively reach people like you with information on tuberculosis? (Check all that apply)	News on Vietnamese social media, including Facebook	152	66.1
	Newspapers and magazines	53	23.0
	TV	42	18.3
	Printed materials from local authorities and medical facilities	69	30.0
	Family, friends, neighbors and colleagues	29	12.6
	At the workplace, place of training of technical interns, or schools	32	13.9
	Others (Free descriptions)**	6	2.6
Which of the following electronic devices do you own? (Check all that apply)	Smartphone (Wi-Fi only)	179	77.8
	Smartphone (Wi-Fi + telephone number)	65	28.3
	Computer	40	17.4
	Tablet computer (e.g., iPad)	20	8.7
	Others	6	2.6

The number of participants was 230. *Among these, 35 participants described the information as symptoms (9), treatment (7), prevention (7), any information (6), medical fees (3), mode of transmission (2), examination (1), and information of support (1) or medical facilities that can provide language support (1). **Among these, 1 participant described the sources as Vietnam festival in Japan

Table 5 presents the results of the multiple logistic regression analysis for potential factors associated with non-health-seeking behaviors among the Vietnamese participants. Participants who believed that TB was incurable were significantly more likely to report non-health-seeking behavior (aORs = 3.12; 95% CI 1.14–8.52) compared to those who believed that TB was curable. In addition, unemployed participants were more likely to exhibit non-health-seeking behaviors (aORs = 58.56; 95% CI 3.85–889.86) compared to those who were employed.

**Table 5:** Multiple logistic regression of the potential factors associated with non-health-seeking behaviors among the Vietnamese participants

Category	Subcategory	Univariate (Unadjusted)				Multivariable aOR			
		OR	95% CI		*p*-value	OR	95% CI		*p*-value
Sex	Male	1.0				1.0			
	Female	1.38	0.59	3.23	0.45	2.11	0.72	6.21	0.18
Age group	18–24	1.0				1.0			
	> 25	0.54	0.23	1.27	0.16	1.03	0.33	3.18	0.96
Occupation	Worker*	1.0				1.0			
	Technical intern trainees	10.71	1.38	83.15	0.02	6.66	0.76	58.05	0.09
	Students**	7.43	0.80	69.05	0.08	6.52	0.56	75.36	0.13
	Others***	5.42	0.32	92.65	0.24	3.67	0.19	69.35	0.39
	Unemployed	65.00	5.12	825.79	0.001	58.56	3.85	889.86	0.003
Health insurance	Yes	1.0				1.0			
	No and others	2.80	0.71	10.98	0.14	2.41	0.49	11.94	0.28
Japanese language proficiency	Intermediate level and higher****	1.0				1.0			
	Basic level and lower*****	1.68	0.72	3.93	0.23	1.95	0.65	5.84	0.24
Do you have any health problems?	No	1.0				1.0			
	Yes	0.42	0.09	1.84	0.25	0.54	0.11	2.71	0.45
Have you ever learned about tuberculosis?	Yes	1.0				1.0			
	No	2.00	0.72	5.58	0.19	1.42	0.45	4.44	0.55
Can tuberculosis disease be cured?	Yes	1.0				1.0			
	No	4.03	1.64	9.89	0.002	3.12	1.14	8.52	0.03
Is there anything to worry about if you are diagnosed with tuberculosis in Japan?	No	1.0				1.0			
	Yes	0.35	0.13	0.93	0.03	0.41	0.14	1.19	0.10

Multivariable models adjusted for sex, and age

aOR, adjusted odds ratio; CI, confidence interval

Among the variables related to knowledge and attitudes towards TB, only those that were statistically significant in the univariate analysis (*p*-value < 0.05) were included in this model. *"Worker" includes office workers, teachers or researchers, and health personnel or long-term care workers. **"Students" includes Japanese language students, and university students (undergraduate or postgraduate). ***"Others" includes self-employed or freelance, household workers, and others. ****"Intermediate level and higher" includes native level, very well (equivalent to N1), well (equivalent to N2), and intermediate (equivalent to N3). *****"Basic level and lower" includes basic (equivalent to N4), and nonfluency in Japanese

In the univariate logistic regression analysis, technical intern trainees were more likely to have non-health-seeking behaviors (ORs = 10.71; 95% CI 1.38–83.15) compared to workers, while participants who were worried about being diagnosed with TB in Japan were less likely to report non-health-seeking behavior (ORs = 0.35; 95% CI 0.13–0.93) compared to those who reported having no concerns. However, this association was not statistically significant in the multiple logistic regression analysis.

## Discussion

Most of the Vietnamese migrants who participated in this study were recent migrants, young adults, and technical intern trainees or workers. They had limited opportunities to learn about TB and had several misconceptions and fears regarding TB, whereas more than half of the participants demonstrated positive attitudes, including compassion towards patients with TB and a willingness to support them.

Although most participants were willing to seek care for TB symptoms, misconceptions about TB treatment were significantly associated with non-health-seeking behaviors. Previous studies have reported that limited knowledge of TB is associated with inappropriate health-seeking practices [18, 19]. The proportion of participants in our study who believed that “TB is incurable” was similar to that reported among migrant and seasonal farmworkers in Ethiopia [20], but higher than the proportion reported among migrants in Jordan [21] and residents in a Vietnam study conducted almost 20 years ago [22], There is a need to strengthen efforts to disseminate knowledge that TB is a curable disease in a way that reaches migrants.

Participants who were unwilling to visit medical facilities if they had TB symptoms mainly cited concerns about medical costs and language difficulties in accessing healthcare services. These factors can hinder migrants from accessing healthcare services [23]. In Japan, if a TB diagnosis is made, public subsidies for TB treatment are available upon application [24]. However, before a TB diagnosis, individuals must pay some of the medical expenses not covered by health insurance, which could be a financial burden, especially for low-income migrants. Half of the unemployed individuals in our study chose not to engage in health-seeking behaviors. Therefore, information on TB for migrants should include information on public subsidies and language assistance to encourage access to healthcare. In addition, a supportive environment is needed to help migrants balance treatment with work or schooling because these social factors are the reasons for their stay in the host country.

Most participants were willing to access healthcare services at various times if they experienced TB symptoms. However, caution is needed concerning the findings, which showed that about a quarter of the participants said, “I would when treatment on my own does not work.” Previous studies conducted in Vietnam [25] and Ethiopia [26] revealed that the major initial responses of patients with TB or persons with presumptive TB after experiencing TB symptoms, including prolonged cough, were to do nothing, self-treat at home, or go to a pharmacy because they thought the symptoms were caused by a cold or non-severe illness. These initial behaviors would result in a delay in accessing appropriate healthcare because of the poor perception of TB risk [25, 27]. In our study, approximately 60% of the participants knew that prolonged coughing was a symptom of pulmonary TB. However, this common symptom of pulmonary TB may complicate the perception of TB risk; thus, a strategy is needed to raise awareness of TB risk.

Migrants’ health-seeking behaviors need to be assessed considering their misconceptions and attitudes towards TB as well as socio-environmental factors [28, 29]. Although not all patients with TB are infectious, most participants believed that persons with TB were infectious. Such misconceptions about TB could lead to fear of other reactions [30, 31]. Many reported limited social support and would only inform their family if diagnosed, likely due to stigma against TB [22, 30]. For example, technical intern trainees, who account for one-third of Vietnamese residents in Japan [9], cannot live with family in Japan [32]. A previous study found that Vietnamese migrants in Japan who relied on health advice from family members in Vietnam or overseas were more likely to have symptoms suggestive of TB compared to those who did not [11]. Emotional support and advice from family members living in their home country are important, however family members may not be familiar with medical information in Japan. Therefore, it is necessary to create an environment in which migrants can seek health consultations, including for TB, in their destination countries.

The findings of this study suggest that tailored TB risk communication needs to be developed by considering the target group's knowledge of TB, especially misconceptions and behavioral characteristics in the destination country. To promote early access to appropriate healthcare and examinations for TB, information for Vietnamese migrants should include the fact that TB is curable with appropriate treatment, the existence of financial and social support for patients who need TB treatment in Japan, and the risk of TB among persons born in a high TB-burden country. Moreover, based on their suggestions, social media and social networking services in migrants’ native languages, should be utilized by health authorities and experts for information dissemination and TB risk communication rather than relying solely on traditional methods, such as printed brochures. Furthermore, TB education must be provided to migrants through various opportunities before and after their migration to Japan.

To our knowledge, this is the first study on TB-related KAP and its association with health-seeking behaviors that specifically targets Vietnamese migrants in Japan using the PAR approach with migrant communities. The findings obtained can be utilized in actual risk communication programs. However, this study had some limitations. First, the sample size was small. Second, the wide confidence interval (95% CI 3.85–889.86) suggested a high degree of uncertainty, which may be due to the small sample size of unemployed people (*n* = 6). Third, selection bias may have occurred because the participants were limited to Vietnamese migrants who attended cultural festivals in these two cities. As this was an exploratory cross-sectional study using purposive sampling, our primary aim was to collect data from more than 200 participants to capture diverse perspectives within a targeted, hard-to-reach population. Given the exploratory nature and logistical constraints of participant recruitment, we did not conduct a priori sample size estimation based on statistical power. Thus, the survey sample was not necessarily representative of Vietnamese migrants living in Japan. Other limitations include recall bias of the participants regarding their responses to health conditions and the validity of the KAP questionnaire. Despite these limitations, this study provides valuable data on TB-related KAP and health-seeking behaviors among migrants, including those with TB-like symptoms. Additionally, migrant volunteers successfully recruited participants using face-to-face approaches, including those who may not normally be interested in TB-related surveys. The participants lived in 19 of the 47 prefectures across Japan.

## Conclusion

The majority of Vietnamese migrant participants had limited opportunities to learn about TB and harbored several misconceptions and fears regarding the disease. Misconceptions regarding TB treatment were significantly associated with non-health-seeking behaviors. Concerns about being diagnosed with TB included social factors related to their reasons for staying in the host country. The findings of this study indicate that tailored TB risk communication for migrants from high-TB-burden countries must be developed to address the target group's misconceptions and concerns regarding TB in the host country. Further efforts are needed to provide migrants with accurate TB knowledge and support in order to help them feel comfortable accessing healthcare. This includes providing TB education, disseminating information before and after migration, and providing language assistance, as well as work- and school-related social support.

## Data Availability

The aggregated data used in this study might be available upon reasonable request.

## Abbreviations

aOR: Adjusted odds ratio
CI: Confidence interval
KAP: Knowledge, attitudes, and practices
OR: Odds ratio
PAR: Participatory action research
TB: Tuberculosis
